# The role of neutrophils in allergic disease

**DOI:** 10.1093/cei/uxae126

**Published:** 2024-12-26

**Authors:** James Trayer, Johana Isaza-Correa, Lynne Kelly, Maeve Kelleher, Jonathan Hourihane, Aideen Byrne, Eleanor Molloy

**Affiliations:** Discipline of Paediatrics, School of Medicine, Trinity College Dublin, Ireland; Discipline of Paediatrics, School of Medicine, Trinity College Dublin, Ireland; Discipline of Paediatrics, School of Medicine, Trinity College Dublin, Ireland; Department of Allergy, Children’s Health Ireland at Crumlin, Dublin, Ireland; Department of Allergy, Children’s Health Ireland at Temple Street, Dublin, Ireland; Paediatrics and Child Health, Royal College of Surgeons in Ireland, Dublin, Ireland; Discipline of Paediatrics, School of Medicine, Trinity College Dublin, Ireland; Department of Allergy, Children’s Health Ireland at Crumlin, Dublin, Ireland; Discipline of Paediatrics, School of Medicine, Trinity College Dublin, Ireland; Department of Neurodisability, Children’s Health Ireland at Tallaght, Dublin, Ireland; Paediatrics, Coombe Hospital, Dublin, Ireland

**Keywords:** polymorphonuclear cell, PMN, granulocyte, atopy, anaphylaxis

## Abstract

Neutrophils are short-lived cells of the innate immune system and represent 50–70% of the circulating leucocytes. Their primary role is antimicrobial defence which they accomplish through rapid migration to sites of inflammation followed by phagocytosis, degranulation, and the release of neutrophil extracellular traps (NETosis). While previously considered terminally differentiated cells, they have been shown to have great adaptability and to play a role in conditions ranging from cancer to autoimmunity. This review focuses on their role in allergic disease. In particular: their role as potential amplifiers of type 1 hypersensitivity reactions leading to anaphylaxis; their involvement in alternative pathways of food and drug allergy; their role in allergic rhinitis and asthma and neutrophil dysfunction in atopic dermatitis. The use of potential biomarkers and therapeutic targets is also discussed with a view to guiding future research.

## Teaser

Neutrophils play a potential amplifying role in food and drug allergy. Neutrophilic inflammation drives a discrete phenotype of treatment-resistant and often severe asthma. Neutrophil dysfunction is a feature of atopic dermatitis contributing to skin flora dysbiosis.

## Graphical Abstract

**Figure:**
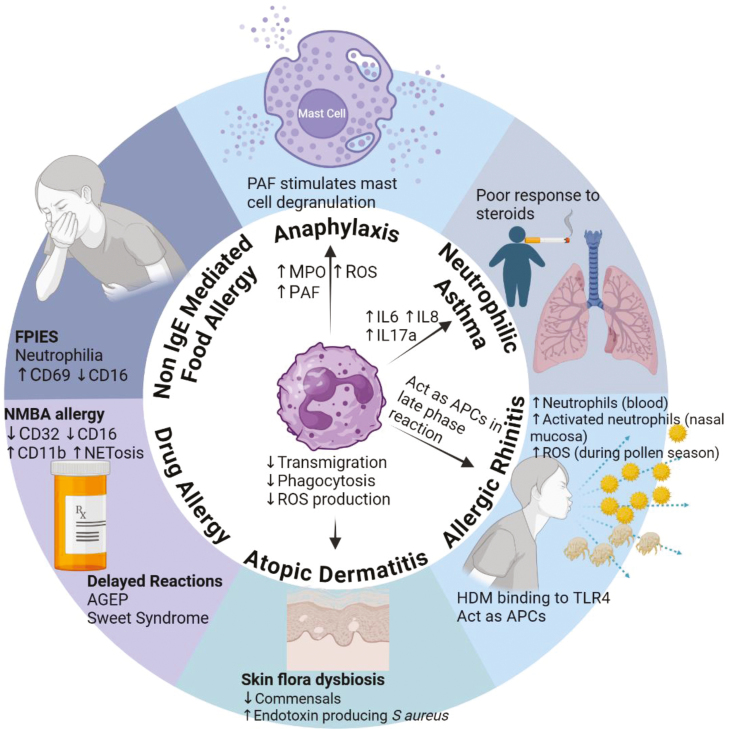
Graphical Abstract- the role of neutrophils in allergic disease Neutrophils play a potential amplifying role in food and drug allergy. Neutrophilic inflammation drives a discrete phenotype of and often severe asthma. Neutrophil dysfunction is a feature of atopic dermatitis contributing to skin flora dysbiosis.

## Introduction

Allergic diseases are amongst the most common chronic diseases worldwide. The prevalence is highest in Western societies though rates are increasing in the rest of the world [1]. In Western Europe, the prevalence of allergic disease among children has been reported as 8.3–31.2% for asthma [2], 9.5–20.2% for allergic rhinoconjunctivitis [2], 4–11.1% for eczema [2], and 3–10% for food allergy [3]. These conditions represent both a significant global health burden in terms of reduced economic output [4] and school performance [5], as well as life-threatening acute illnesses such as anaphylaxis and severe asthma.

While allergic diseases are typically considered disorders of the adaptive immune response there is evidence that the innate immune system also plays a role. This includes skin barrier defects such as filaggrin mutations leading to increased rates of eczema and subsequent food and aeroallergen sensitization [6]. In addition, epithelial-derived cytokines such as Thymic stromal lymphopoietin (TSLP) act on multiple cell lineages including neutrophils, mast cells, basophils, and type two innate lymphoid cells (ILC2). TSLP acts directly on dendritic cells which in turn promotes the differentiation of naïve CD4+ T cells into TH2 cells producing interleukin 4 (IL-4), IL-5, and IL-13 [7, 8]. TSLP plays an important role in the pathophysiology of asthma with increased levels associated with more severe disease [9]. Tezepelumab is an anti-TSLP monoclonal antibody licenced for the treatment of severe refractory asthma and has been shown to reduce exacerbations and improve lung function in this cohort [10].

**Figure 1:**
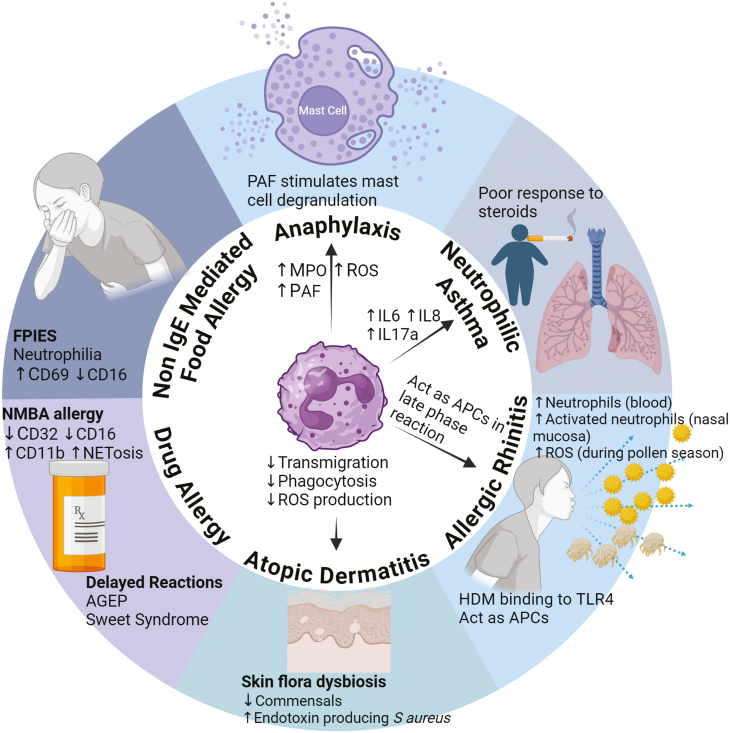
summary of the role of neutrophils in allergic conditions. Created with Biorender.com

Neutrophils are key cells of the innate immune response to infection. They are the most abundant leucocyte in the circulation and migrate to sites of infection and inflammation. They are stimulated by a variety of different cytokines including TSLP. In this article, we review the role of neutrophils beyond their antimicrobial function in all forms of allergic disease (Fig. 1).

## Neutrophil function and activation

Neutrophils are produced in the bone marrow in large numbers from haematopoietic stem cells, approximately 10^11^ daily, before circulating around the body [11]. They are key players in the innate immune system and represent 50–70% of circulating leucocytes [12]. Neutrophils are recruited to tissues in response to cytokines such as IL-8, IL-17, CXCL1, and CXCL2 [13] and transmigrate from the blood vessels into the tissue via the leucocyte adhesion cascade [14]. In tissue, their primary role is killing pathogens through phagocytosis, the release of reactive oxygen species (ROS), and the release of neutrophil extracellular traps (NETosis) [15]. Markers of neutrophil activation include the presence of ROS produced during the oxidative burst as well as myeloperoxidase (MPO) which is present in azurophilic granules within neutrophils and released into phagosomes during phagocytosis [16]. Neutrophils also rapidly produce platelet-activating factor (PAF) when activated. PAF is a potent vasoactive mediator causing vasodilation, platelet aggregation, and bronchoconstriction. PAF plays an important role in both sepsis and anaphylaxis [17, 18].

Neutrophils typically live for less than 24 h once they leave the bone marrow for circulation [19]. In sites of inflammation, neutrophils are activated and survival is enhanced by cytokines including granulocyte colony-stimulating factor (G-CSF), granulocyte-macrophage colony-stimulating factor (GM-CSF), tumour necrosis factor alpha (TNFα), and lipopolysaccharide (LPS) [20]. In tissues, neutrophils are the first immune cells to migrate to sites of infection/inflammation [21] and modulate the subsequent immune response by selective cytokine production [22]. Furthermore, they act as antigen-presenting cells, presenting phagocytosed antigen to local T cells which modulates the adaptive immune response [23, 24].

Neutrophils are terminally differentiated cells, though it has long been recognized that they are not a homogenous group and that different subpopulations play many additional roles [11]. Various cell types have been described based on microscopic appearance and cell surface markers though it has been suggested that the ability of neutrophils to adapt to the local environment may be responsible for these differences rather than genuine distinct subpopulations [25].

## Pathophysiology of allergic reactions

Allergic reactions are characterized by an immune-mediated response to a typically harmless antigen. There are various types of reactions that differ in timing, immunological mediators, and severity of reaction. In brief, three types of immune response are described though there may be overlap and interaction between the cells involved in each type. Type 1 reactions are directed towards intracellular pathogens and involve type 1 T helper cells (Th1 cells), type 1 innate lymphoid cells (ILC1), natural killer cells (NK), and cytotoxic CD8 + cells. There is activation of innate cells such as macrophages and neutrophils. Type 2 reactions are directed towards large extracellular pathogens such as helminths. Type 2 T helper cells (Th2 cells) and ILC2 cells, mast cells, basophils, and eosinophils are the main cells. IL-4, IL-5, IL-9, and IL-13 are the main cytokines. Type 3 reactions target extracellular bacteria and fungi. They are directed by Th17 cells, type 3 innate lymphoid cells (ILC3) with the production of IL-17 and recruitment of neutrophils as the main effector cells.

The classic Gell and Coombs classification has been refined as our understanding of the underlying immunological mechanisms has increased [26, 27]. The immediate type I hypersensitivity reaction is mediated primarily by mast cells. In a sensitized individual, antigen binds to allergen-specific IgE attached to FcεRI on the surface of mast cells. This leads to receptor cross-linking and degranulation with the release of multiple preformed mediators such as histamine, tryptase, and PAF. Anaphylaxis is the most severe form of type I hypersensitivity.

Type II reactions are antibody-dependent. Circulating IgG or IgM binds to antigen on the surface of the body’s own cells leading to immune mediated cell destruction. Autoimmune conditions such as autoimmune haemolytic anaemia and Grave’s disease are examples of type II reactions.

Type III reactions involve circulating IgG binding to soluble antigens forming an immune complex that can then deposit in various tissues leading to localized inflammation with activation of neutrophils via FcγRs and activation of the complement system. Serum sickness and post-streptococcal glomerulonephritis are both type III reactions.

Type IV reactions are mediated by T cells and are typically delayed. Examples include contact dermatitis and the severe cutaneous adverse reactions (SCARs) seen in some drug allergies such as Steven Johnson Syndrome. This group is further subdivided based on the immune response [27]. Type IVa feature a type 1 immune response with the involvement of Th1 cells and interferon-gamma (INF-γ) as the primary mediator. Type IVb is predominantly a type 2 immune reaction with Th2, ILC2s, and eosinophils as the primary cells involved. IL-4, IL-5, and IL-13 are the main cytokines. Clinical manifestations of IVb reactions include allergic rhinitis, asthma, and atopic dermatitis. Type IVc reactions are predominantly type 3 reactions with Th17 and ILC3 producing IL-17 and IL-22. ILC3 cells produce IL-8 which recruits neutrophils from the circulation leading to inflammation and tissue damage as seen in neutrophilic asthma.

Type V reactions are characterized by epithelial barrier dysfunction resulting in penetration of environmental pollutants, allergens, and microbes triggering epithelial cell production of alarmins, IL-33, IL-25, and TSLP.

Type VI reactions recognize the impacts of obesity on immune function. Clinically this manifests as an asthma phenotype characterized by obesity, adult-onset of symptoms, and increased airway innate immune system activation.

Type VII reactions involve direct activation of immune cells by chemicals including drugs such as non-steroidal anti-inflammatories (NSAIDs), neuromuscular blocking agents, opioids, and antibiotics.

## Neutrophils as potential amplifiers of the immune response during anaphylaxis

Anaphylaxis is defined as a life-threatening reaction characterized by acute onset of symptoms involving different organ systems and requiring immediate medical intervention [28]. It is most commonly triggered by systemic release of pre-formed histamine, from mast cells, and activated via the cross linking of antigen bound FcεRI [29]. Mast cell degranulation can also be initiated via IgG/FcγRI, activation of complement cascade, TLR activation, and via MRGPRX2 [30].

The classic features of anaphylaxis are caused by vasodilation and increased vascular permeability leading to extravascular fluid leak into subcutaneous and submucosal tissues. This leads to rapidly progressive circulatory and respiratory compromise usually accompanied by widespread urticaria and angioedema. The incidence rate of anaphylaxis has been reported as 0.14 per 100 person years with higher incidence in young children [31]. Reaction severity is unpredictable, and people can experience systemic reactions of differing severity, to apparently similar allergen exposure. Influencers of severity are known collectively as “cofactors” and include exercise, illness, and NSAIDs. The mechanism behind their effect is not fully defined.

### Neutrophil activation in anaphylaxis

Systemic allergic reactions are associated with an increase in blood neutrophils which correlates with reaction severity and reaction threshold [32–35]. Neutrophils are activated during anaphylaxis with reduced soluble CD62L (also known as L-selectin, a protein involved in neutrophil migration) and raised MPO compared to healthy controls [36]. MPO is secreted by activated neutrophils as part of NETosis [37]. MPO levels were 2.9-fold higher in moderate and 5 times higher in severe anaphylaxis. MPO levels were raised in all cases of anaphylaxis regardless of whether there was a detectable rise in histamine or tryptase.

Genomic studies have demonstrated the upregulation of genes associated with the innate immune response during anaphylaxis with a similar pattern to that seen in response to LPS [38]. A collection of six genes associated with acute-phase response and pro-inflammatory processes have been identified as key drivers of anaphylaxis in humans, specifically PADI4, IL1R2, PPP1R3D, KLHL2, ECHDC3, and LTB4R [32]. A similar pattern was observed in patients presenting with anaphylaxis to the emergency department or during food challenge [33]. Transcriptomic studies in humans and mice during the acute allergic response identified upregulation of genes involved in innate immune function, cellular stress, and apoptosis with a group of 11 genes correlating with reaction severity including innate immune system genes CLEC4A, IL18R1, IFNGR1, CD59B, and GPR27 [39]. Transcriptomic studies of children with peanut allergy experiencing reactions demonstrated increased transcription of genes associated with B-cell activation, TLR signalling, and stimulatory FcγR-mediated phagocytosis [35].

Neutrophils are the main source of clinically important mediators such as PAF and MPO. As neutrophils are the most numerous leucocyte in circulation and mast cells are restricted to tissues, they may serve as amplifiers of the initial mast cell response. This may explain how exposure to allergen in one site such as the gut can rapidly produce symptoms of anaphylaxis involving the skin, respiratory and circulatory systems.

### Experimental data from humans and murine models

**Figure 2:**
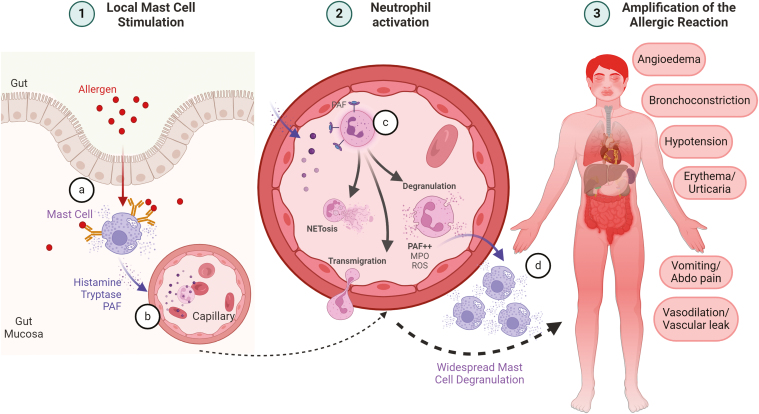
the potential amplifying role played by neutrophils in anaphylaxis. (**a**) Allergen binds to the surface of mast cells with release of preformed mediators, (**b**) these mediators enter the circulation, (**c**) neutrophils in circulation are activated by mast cell released mediators leading to NETosis, transmigration and degranulation, (**d**) neutrophil produced mediators such as PAF lead to further mast cell degranulation and systemic symptoms. Created with Biorender.com

Evidence for this theory is small but includes data from both human and murine models. PAF has been suggested as a potential amplifying signal as once secreted by neutrophils it can cause degranulation of mast cells (Fig. 2) [40, 41]. Similar neutrophil derived pro-inflammatory signalling is seen in cytokine storm and in severe cases of SARS-CoV-2 infection [42]. Intercurrent infections are known to increase severity of allergic reactions [43]. If neutrophils are acting to amplify the allergic response then pre-activation by infection may increase this effect contributing to more severe symptoms. Finally, neutrophils express both the FcεRI [44] and several FcγRs [45] and could therefore be stimulated directly by allergen independent of mast cells.

Anaphylaxis is unpredictable and potentially life-threatening. Therefore, much of the mechanistic work has been performed in animal models. These studies are useful but it should be remembered that there are challenges in translating findings to humans [46]. The expression of antibody receptors on immune cells differ between species. Laboratory mice are raised in an environment with limited exposure to allergens and other environmental factors that may modulate the immune response. Recognizing anaphylaxis in mice is challenging due to their size with indirect signs such as hypothermia, decreased activity, and diarrhoea used as surrogate markers of cardiac output and vascular hyper-permeability. Despite these challenges, there is some evidence for the role of neutrophils in food allergy reactions. Neutrophil-depleted mice experienced less severe anaphylaxis with a particular reduction in late-phase hypothermia [47–49]. In addition, mice depleted of mast cells and basophils could still develop anaphylaxis with higher levels of PAF post anaphylaxis suggesting neutrophils to be the main cell mediator in this case [47]. Injection of PAF into mice has been shown to mimic anaphylaxis [50]. In humans, PAF levels are raised in anaphylaxis and correlate with disease severity [17]. Luminescence, used as a marker of MPO presence, is detectable within minutes of antigen challenge in mice [49]. *In vivo* studies demonstrated activation of both basophils and neutrophils when whole blood was incubated with peanut extract [51]. These experiments suggest that neutrophils may be directly activated by the inflammatory cascade initiated by allergen encounters. There is an alternative anaphylaxis pathway in mice in which allergen can directly stimulate neutrophils by forming antigen-IgG complexes and binding to stimulatory FcγRs on the surface of neutrophils [49]. As these receptors have a lower affinity for allergen than the FcεRs, a higher concentration of allergen is required and therefore intravenous challenge is required to trigger a reaction [52].

Neutrophil activation may also be dependent on platelet function. Both neutrophils and platelets expressing the FcγRIIa (CD32a) and transgenic mice expressing FcγRIIa demonstrated platelet activation by IgG-antigen complexes along with a reduction in platelet count following anaphylaxis. Platelet depletion served to reduce the severity of anaphylaxis while symptom severity was increased in mice with thrombocytosis [53]. Platelet aggregation on neutrophils has been shown to increase the production of ROS and NETosis [54]. This study also identified activated platelets aggregating with 80% of circulating neutrophils during anaphylaxis [53]. As platelet numbers in circulation are much higher than neutrophils it is possible that IgG-antigen complexes activate platelets initially and that their aggregation on neutrophils leads to neutrophil activation.

### Neutrophils in lipid transfer protein allergy

Lipid transfer proteins (LTP) are a group of highly conserved structurally similar proteins that are present in fruits, vegetables, nuts, and pollens [55]. LTP allergy is particularly common in Southern Europe where it is the most common trigger for anaphylaxis [55, 56]. A feature of LTP allergy is the significant variability in reaction phenotype. This can range from contact urticaria, and oral-allergy syndrome through to anaphylaxis [57]. Many patients with LTP allergy require the presence of a co-factor to trigger a reaction (such as NSAIDs and exercise) [57]. While LTP allergy is mediated via the classical IgE-mast cell axis there is some evidence for the role of neutrophils. A study comparing patients with anaphylaxis to LTP without co-factors (LTP-A) to patients with co-factor-dependent allergy identified altered expression of genes involved with the innate immune system [58]. Patients with LTP-A had increased expression of genes related to neutrophil function as well as an increased baseline reactive oxygen species suggesting baseline immune dysfunction [58]. The same study also demonstrated increased neutrophil expression of the FcγRI (CD64) and reduced FcεRI in patients with LTP-A compared to those requiring co-factors. Increased FcγRI was accompanied by increased LTP-specific IgG1 and IgG3. These findings point to a possible mechanism for direct human neutrophil stimulation by allergens, as seen in mouse models [48, 59].

While the exact mechanism remains unclear, it is evident that neutrophils are activated during anaphylaxis with the release of clinically important mediators which may in turn augment the allergic reaction.

## Neutrophils in non-IgE mediated gastrointestinal food allergy

The previous section discussed the role of neutrophils in IgE mediated food allergies. This is the most common allergic phenotype with milk, egg, and peanut being the most commonly implicated foods. However, other forms of food allergy are non-IgE mediated and predominantly affect the gastrointestinal tract. This is a heterogenous group of conditions that typically present in infancy.

Food protein-induced enterocolitis syndrome (FPIES) typically presents in the first year of life. It is the most common non-IgE-mediated food allergy [60]. There is a lack of detectable IgE antibodies to the food trigger and while the reaction is food-specific, the immune response does not appear to involve mast cell activation. Symptoms are primarily gastrointestinal with profuse vomiting which may be followed by diarrhoea but can be more severe with associated pallor, floppiness, dehydration, acidosis, hypothermia, and may progress to hypovolaemic shock [61]. In contrast to IgE-mediated reactions, there are none of the hallmarks of histamine release; urticarial eruptions, angioedema, and respiratory compromise. Raised tryptase levels are not seen. The time kinetics contrast with IgE allergy, with reactions starting 1–4 h after ingestion. Diagnosis is based on clinical presentation and consensus criteria as no specific biomarker exists [61], though neutrophilia has long been recognized as a consistent though non-specific feature of FPIES reactions [62]. As well as an increase in circulating neutrophils, FPIES reactions are also associated with markers of neutrophil activation including increased CD69 and downregulation of CD16 (FcγRIII) as well as increased expression of genes associated with an innate immune response [63]. Circulating lymphocytes are reducing during FPIES reactions [63].

In contrast to FPIES, blood neutropaenia and eosinophilia are associated with disease severity in patients with food protein-induced allergic proctocolitis (FPIAP) [64]. Rectal biopsies demonstrate eosinophilic and neutrophilic infiltration [65, 66]. Food protein-induced enteropathry (FPE) is not associated with either neutropenia or neutrophilia. Coeliac disease, while not typically considered an allergy, shares many clinical features with FPE though is easily distinguished by the presence of IgA anti-tissue transglutaminase antibodies. Coeliac disease is often associated with duodenal neutrophilia, especially in children, and this correlates with disease activity [67].

## Neutrophils in allergic rhinitis

Allergic rhinitis is a Th2-mediated immune condition in which exposure to inhaled allergens such as grass pollen leads to an immediate immune response with the release of preformed mediators from mast cells. These mediators cause rhinorrhoea, sneezing, itchy, and watery eyes. The immediate phase response leads to increased expression of cellular adhesion molecules and an influx of eosinophils, neutrophils, and macrophages which are responsible for the late phase reaction with local inflammation leading to nasal congestion [68]. Patients with allergic rhinitis have increased levels of neutrophils in circulation as well as nasal mucosa and nasal lavage fluid compared to healthy controls when measured during the pollen season [69]. There is a higher proportion of activated neutrophils (CD16^high^ CD62L^dim^) within the nasal mucosa and these demonstrate a potential ability to both activate CD4+ T cells and enhance eosinophil migration [69]. Other studies have confirmed that neutrophils play a role as antigen-presenting cells (APCs) and can induce proliferation of allergen-specific T cells involved in the late-phase response [23, 24]. Immunotherapy to inhaled allergens is associated with a reduced neutrophil infiltrate in late-phase response when measured in a skin chamber model [70]. This is seen as early as six months into the treatment course. The late-phase response is also reduced in nasal mucosa and the lung following immunotherapy [71].

House dust mite (HDM) allergy is one of the most common causes of allergic rhinitis. HDM faeces and body fragments are present in household dust along with other potential irritants such as fungal spores, bacteria, and environmental pollutants. This combination has the potential to activate the innate immune system with the production of inflammatory cytokines such as GM-CSF, IL-25, IL-33, and TSLP, leading to subsequent Th2 predominant inflammation [72]. HDM components including Der p2, Der f2, and Der p38 have been shown to bind to TLR4 receptors and lead to neutrophil activation. In mouse models, injection of Der p38 led to an increase in both neutrophils and eosinophils in the lung while in TLR4 knock-out mice there was no change in cell counts [73]. Der p38 was also found to delay neutrophil apoptosis in both healthy and allergic subjects [73].

In patients with HDM allergy, exposure to the allergen inhibits neutrophil apoptosis via lymphocyte-produced cytokines [74]. *In vitro* studies of human neutrophils demonstrated delayed neutrophil apoptosis when incubated with *Dermatophagoides pteronissinus* extract via activation of multiple pathways including TLR4 and NF-kB and suppression of the caspase 9/3 pathway [75].

The primary allergenic component in Timothy grass pollen is Phl p1 and is conserved across grass species. Incubation of murine respiratory epithelial cells with Phl p1 resulted in increased secretion of proinflammatory cytokines IL-6 and IL-8, both of which promote neutrophil migration [76]. In patients with an allergy to cock’s foot grass pollen (*Dactylis glomerata*), neutrophils are activated when incubated with allergen extract in a dose dependent fashion [77]. This effect is markedly reduced in patients treated with a course of allergen-specific immunotherapy. Oxidative stress has been demonstrated in patients with allergic rhinitis (both with and without co-existing asthma) when measured during the pollen season [78].

Efforts have been made to use leucocyte ratios as biomarkers for allergic rhinitis severity. In children, an increased ratio of neutrophils to lymphocytes (NLR) was shown to be associated with both a diagnosis of allergic rhinitis and to correlate with disease severity [79, 80]. In adults, however, there is a decreased NLR with an increase in eosinophil-lymphocyte ratio (ELR) which is more pronounced in those with persistent symptoms [81]. These conflicting results make the use of cellular components of blood, as a biomarker of allergic rhinitis, challenging. However, it is clear that neutrophils play a role in allergic rhinitis and are involved in priming the adaptive immune response in the late phase response.

## Neutrophilic asthma

Asthma is one of the most common chronic diseases and is estimated to affect 358 million people worldwide [82]. There is no single diagnostic test for asthma and diagnosis is based on a history of recurrent respiratory symptoms such as wheeze and shortness of breath accompanied by evidence of reversible airway obstruction [83].

Asthma is a heterogenous disease and different phenotypes exist. In contrast to the more common eosinophilic asthma, there is a cohort of patients in whom neutrophils are the predominant inflammatory cell in sputum. These patients are typically older with late-onset disease, are male and have more severe disease with more frequent hospitalizations and higher doses of prescribed corticosteroids [84, 85]. Neutrophilic asthma is less likely to be associated with other atopic conditions. Neutrophilic asthma also predominated in a group of patients with sudden-onset fatal asthma [86]. Neutrophilic asthma represents 20–30% of cases of asthma globally though epidemiological studies demonstrate significant geographic variability with rates varying between 5% in Greece to 59% in India [84, 87, 88].

It is unclear why some people develop a neutrophilic phenotype of asthma though contributing factors have been identified. The host's response to infection or environmental pollutants may trigger the initial inflammation. A Canadian study found that people living close to a major road had higher rates of asthma diagnosis particularly neutrophilic asthma [89]. This may also explain the high rates seen in India where air pollution is among the worst in the world [90]. Similarly, cigarette smoke has been shown to result in increased IL-17a, IL-6, and IL-8 as well as increased neutrophils in the airway mucosa of people with asthma [91]. Intrinsic host factors may also play a role. Obesity is known to be an inflammatory state [92] and to be associated with the development of asthma [93]. BMI is positively associated with increasing sputum neutrophil counts and a higher proportion of obese females had neutrophilic asthma compared to control with normal BMI [94]. Studies have shown that neutrophil function changes with age [95]. In addition, the neutrophils of people with asthma have been shown to have increased migration and NETosis, reduced phagocytosis, and increased proinflammatory cytokines such as CXCL-8 and IL-1β [95].

While specific cut-offs used for the neutrophil count in sputum vary [95], the existence of a neutrophil-predominant phenotype in asthma is clear. In contrast to other allergic conditions where neutrophils may act to amplify the allergic response, neutrophilic asthma represents a distinct form of asthma in which environmental and intrinsic factors combine leading to airway inflammation.

## Neutrophil dysfunction in atopic dermatitis

Atopic dermatitis is a chronic skin condition characterized by dry, itchy, and inflamed skin predominantly affecting the flexures and associated with other atopic conditions [96]. It usually first presents in infancy and affects up to 20% of children though there is significant geographical variability [97]. Risk factors for the development of atopic dermatitis include intrinsic factors such as loss of function mutations in FLG, the gene encoding for filaggrin. Filaggrin is a key protein involved in the formation of the stratum corneum of the skin. Loss of filaggrin leads to reduced skin barrier integrity, increased water loss, and a predisposition to develop eczema [98]. Extrinsic factors such as urban living, smaller family size, and early antibiotic exposure are also associated with an increased risk of atopic dermatitis [99].

Atopic dermatitis is associated with dysbiosis of the skin flora with an increase in *Staphylococcus aureus* and a decrease in commensals [99]. The skin of almost all adults with atopic dermatitis is colonized with *S aureus* with up to 65% strains producing exotoxins [100]. Despite the presence of toxin-producing bacteria there is a marked paucity of neutrophils seen in skin biopsies of patients with atopic dermatitis, even during acute infection [101]. Migration of neutrophils from the circulation to the skin is impaired in atopic dermatitis [101] with reduced chemotactic response, phagocytosis, and reduced ROS production [102–105]. Neutrophils recognize pathogens via TLRs and a cohort of patients with atopic dermatitis have polymorphism of TLR2 which is associated with severe disease phenotype [106]. It is likely that neutrophil dysfunction in atopic dermatitis stems from impaired epithelial cell signalling such as decreased LL37 as patients with atopic dermatitis are not at increased risk of infections at other sites as would be expected in generalized neutrophil defects [107].

## Neutrophils in drug allergy

Drug allergy encompasses many different immune-mediated adverse reactions to medications. They are frequently divided into immediate and delayed hypersensitivity reactions. Immediate hypersensitivity reactions are type I mast cell-mediated and typically happen within 1–2 h of exposure. Mast cell activation in the context of drug allergy is not exclusively through FcεRI. There are multiple proposed pathways of mast cell activation for different drugs. In the case of neuromuscular blocking agents (NMBAs), IgE antibodies are detected in most cases of anaphylaxis. NMBA activation of mast cells via MRGPRX2 has also been proposed. Patients with NMBA allergy have also been shown to have higher levels of circulating anti-NMBA IgG and levels correlated with reaction severity [108]. During anaphylaxis they had reduced expression of FcγRIIa and FcγRIII suggesting binding by IgG-antigen complexes. This was accompanied by markers of neutrophil activation including raised CD11b, neutrophil elastase, and NETosis. PAF-AH levels were inversely related to reaction severity [108]. This suggests amplification of the allergic reaction due to neutrophil activation.

Delayed-type hypersensitivity reactions occur hours to weeks after exposure to the culprit drug. Delayed hypersensitivity reactions are T-cell mediated although neutrophils can play a role in some phenotypes. Acute generalized exanthemous pustulosis (AGEP) is most often triggered by antibiotics though may also be triggered by other drugs and viruses [109]. Drug-specific CD4 T cells produce IL-8 and GM-CSF which lead to neutrophil chemotaxis and activation. It is hypothesized that CD8 cytotoxic T cells kill keratinocytes leading to vesicle formation with neutrophils causing progression to pustules. Skin biopsy shows a neutrophil-predominant infiltrate [109]. Sweet syndrome (acute febrile neutrophilic dermatosis) is characterized by tender erythematous papules and plaques, fever, and may have associated joint and eye involvement. Skin biopsy demonstrates neutrophilic infiltration [109]. Sweet syndrome may be triggered by infection, malignancy, or medications.

Drug-induced enterocolitis syndrome (DIES) is an uncommon form of drug allergy most commonly triggered by amoxicillin. It presents 2–4 h after drug administration with vomiting, abdominal pain, diarrhoea, pallor, lethargy, and may lead to hypovolameic shock. It is associated with neutrophilic leukocytosis [110]. The underlying pathophysiology is unknown but the presentation shares many clinical characteristics with FPIES suggesting possible shared pathways.

## Potential biomarkers and therapeutic targets

Predicting which patients with food allergies will develop severe anaphylaxis is challenging and there are no reliable biomarkers [43]. Biomarkers would be particularly useful in patient selection as food allergy management moves into the age of oral immunotherapy. All myeloid cells can secrete PAF including mast cells, basophils, and macrophages though neutrophils are by far the most numerous PAF-secreting cells [111]. In anaphylaxis, PAF levels have been found to correlate with reaction severity better than either histamine or tryptase and therefore represent a potential biomarker for anaphylaxis [17]. Similar results have been found in a prospective study of children presenting to the emergency department with anaphylaxis [112]. In humans, PAF levels during anaphylaxis are inversely proportional to PAF-AH levels and were lowest in patients with fatal anaphylaxis suggesting PAF-AH levels may be an independent determinant of anaphylaxis outcome as a reduced capacity to metabolize PAF increases the allergic response [41, 112]. In mouse models, PAF knock-out mice had significantly reduced anaphylaxis severity compared to wild-type mice [113]. PAF-AH levels could potentially be used to identify patients at higher risk of severe anaphylaxis.

The PAF receptor (PAFR) could also represent a potential therapeutic target. While there are challenges in translating from murine models of anaphylaxis to humans, PAF antagonists significantly attenuated the severity of anaphylaxis in mice while histamine blockade had no effects [114]. Combined PAF and histamine blockade was most effective in preventing anaphylaxis. Some antihistamines have been shown to have anti-PAF effects including azelastine, rupatadine, oxatomide, ketotifen, and epinastine, with rupatadine well studied as a treatment for allergic rhinitis [115]. No studies have been performed comparing rupatadine or other PAF antagonists to non-PAF-blocking antihistamines such as cetirizine for food allergic reactions [116].

Neutrophils remain a challenging therapeutic target due to their importance as first responders of the innate immune system and the risks of increased bacterial infections if their function is impaired. Despite this, several trials are ongoing of agents that either directly or indirectly target neutrophils [42]. In asthma, several biologics including dupilumab and tezepelumab are now suggested as potential therapeutic agents for adults and adolescents with neutrophilic asthma [83].

## Knowledge gaps and future research

The innate immune system, and neutrophils in particular, have a role to play in allergies to foods and drugs, allergic rhinitis, asthma, and atopic dermatitis. There remain gaps in our knowledge that warrant future research. The traditional focus of acute type 1 hypersensitivity reactions has been on mast cells (and to a lesser extent basophils) releasing mediators such as histamine. Treatment of reactions is primarily with antihistamines for mild reactions and adrenaline for anaphylaxis with bronchodilators and steroids playing a supporting role. It is clear that type 1 hypersensitivity reactions involve additional cells including neutrophils and platelets [51] and that mediators such as PAF are potentially useful biomarkers [17, 41, 112]. Further work to identify the interactions between cells may reveal other useful biomarkers or therapeutic targets such as PAF antagonists.

PAF-AH activity has been shown to correlate with reaction severity in patients presenting to the emergency department [112]. Larger, prospective studies are required to investigate whether baseline PAF-AH activity could be useful prospectively as an independent risk factor for severe reactions.

As neutrophils may play a role in amplifying the allergic response, research should focus on the pathways leading to their activation. There are several possibilities including antigen-IgG complexes binding to FcγRs as seen in mouse models, initial PAF release by mast cells and basophils activating neutrophils or direct activation by allergen binding to PRRs on the surface of neutrophils similar to HDM proteins binding to TLR4 [73]. Identification of these pathways could lead to targeted therapies that minimally impact neutrophils’ primary role in antimicrobial defence.

## Conclusion

Neutrophils are the most abundant leucocyte in circulation and represent one of the immune system’s first lines of defence against infection. They exert potent antimicrobial effects through phagocytosis, oxidative burst, and NETosis which can lead to localized inflammation and tissue damage. Their abundance, ability to react to a wide variety of stimuli, and their potency as producers of cytokines lead to their involvement in a wide range of clinical conditions including allergy.

Recent studies have shown that neutrophils play a role in a wide range of different pathways and clinical conditions and are no longer considered simple terminally differentiated cells with a single role in fighting bacterial infections. In the field of allergy, there is evidence for neutrophil involvement in many conditions though knowledge gaps remain highlighting that the complexity of allergic mechanisms is poorly understood. It remains unclear the exact role neutrophils play in anaphylaxis in humans. While neutrophils are activated during allergic reactions it remains unknown whether this is via PAF or other mediators released by mast cells, basophils, or via IgG-antigen complexes binding to FcγRs on neutrophils as has been described in mice. Once activated, what role do these neutrophils play in the allergic reaction, and should they be considered amplifiers of the initial mast cell-mediated response? Perhaps conditions such as FPIES, DIES, and neutrophilic asthma are better considered to be primarily inflammatory rather than allergic in nature.

Further research into the role of neutrophils in allergic diseases is required in order to identify potential new biomarkers and therapeutic options.

## Abbreviations

AGEP: acute generalised exanthemous pustulosis
APCs: antigen presenting cells
DIES: drug induced enterocolitis syndrome
ELR: eosinophil to lymphocyte ratio
FcεRI: the high affinity IgE receptor
FcγR: IgG receptors
FPE: food protein induced enteropathy
FPIAP: food protein induced allergic proctocolitis
FPIES: food protein induced enterocolitis syndrome
G-CSF: granulocyte colony stimulating factor
GM-CSF: granulocyte-macrophage colony stimulating factor
HDM: house dust mite
IgA: immunoglobulin A
IgE: immunoglobulin E
IgG: immunoglobulin G
IgM: immunoglobulin M
ILC: innate lymphoid cell
IL-4: Interleukin 4
INF-γ: interferon gamma
LPS: lipopolysaccharide
LTP: lipid transfer proteins
MPO: myeloperoxidase
MRGPRX2: Mas-related G-protein coupled receptor member X2
NET: neutrophil extracellular traps
NK cells: natural killer cells
NLR: neutrophil to lymphocyte ratio
NMBA: neuromuscular blocking agent
NSAID: non steroidal anti inflammatory
PAF: platelet activating factor
PAF-AH: platelet activating factor acetylhydrolase
PAFR: platelet activating factor receptor
PRRs: pattern recognition receptors
ROS: reactive oxygen species
SCAR: severe cutaneous adverse reaction
Th1: type 1 T helper cells
Th2: type 2 T helper cells
TLR4: toll like receptor 4
TNFa: tumour necrosis factor alpha
TSLP: thymic stromal lymphopoietin
